# Diagnostic Challenges of Upper Gastrointestinal Tract Crohn’s Disease

**DOI:** 10.7759/cureus.82221

**Published:** 2025-04-14

**Authors:** Sruthi Beladev, Krupa A Joseph, Nikhil Charles Madathiparambu

**Affiliations:** 1 Acute Medicine, Queen Elizabeth The Queen Mother Hospital, Margate, GBR

**Keywords:** crohn’s disease (cd), endoscopy, histology, nutrition, upper gastrointestinal tract

## Abstract

Crohn's disease (CD) is a chronic inflammatory disorder that commonly affects the distal small bowel and proximal large bowel. However, it can involve any part of the gastrointestinal tract (GIT), including the upper GIT. However, upper GIT involvement is often overlooked due to the limited use of upper gastrointestinal (UGI) endoscopy and biopsy for diagnosis. We present a case of a 30-year-old male with an 11-year history of symptomatic CD. Despite multiple normal findings on various endoscopic and imaging evaluations, his symptoms persisted, leading to numerous treatment modifications from Pentasa foam enema to various biologics. Despite this, fecal calprotectin remained elevated. A critical diagnosis was made via video capsule endoscopy, revealing duodenitis and aphthous ulcers, and subsequent esophagogastroduodenoscopy (OGD) with histology confirming focal inflammation consistent with upper GIT CD of the stomach and duodenum. Despite aggressive treatment, the patient’s symptoms persisted, necessitating comprehensive nutritional management. This case underscores the diagnostic challenges of upper GIT CD and highlights the importance of thorough endoscopic and histological evaluations for accurate diagnosis and effective management. Additionally, it underscores the usefulness of fecal calprotectin as an indication of inflammation and the relevance of nutritional management in controlling symptoms of upper GIT CD.

## Introduction

Crohn’s disease (CD) is a chronic inflammatory condition that can affect any part of the gastrointestinal tract (GIT), from the mouth to the anus. However, it has a predilection for the distal small bowel and proximal large bowel. The inflammation in CD can be discontinuous, affecting multiple segments of the GIT and involving all layers from the mucosa to the serosa [1]. Upper gastrointestinal (UGI) involvement in CD, classified as L4 (UGI) in the Montreal classification (used to classify the severity of ulcerative colitis and CD; in this classification, CD is classified by age of onset, location, and behavior [2]), is relatively rare.

The occurrence of UGI involvement in patients with CD is minimal when considering pathology located proximal to the terminal ileum. However, this estimate may be too low, since in some countries and hospitals, OGD is not commonly performed on CD patients, especially the patients with a long history of CD [3].

Ileocolonoscopy is the primary diagnostic tool for CD, but according to the European Crohn’s and Colitis Organization (ECCO) consensus, it is recommended to also examine the entire small bowel to assess the extent of UGI involvement [3].

This case report presents one such incident of upper GIT CD.

## Case presentation

We present a case of a 30-year-old gentleman with an 11-year history of CD, first diagnosed in 2011 after presenting with per rectal (PR) bleeding, intermittent loose stools, and abdominal pain. Initial colonoscopy only showed anorectal hemorrhoids, but hemorrhoidectomy was not done due to small size (hemorrhoidectomy was later performed in 2024).

The patient continued to be symptomatic, so in 2017 another colonoscopy was done, which showed quiescent proctitis with normal biopsies from the cecum. He was provisionally treated with Pentasa foam enema for proctitis. However, it was noted that the rectal findings did not fully explain his symptoms of persistent PR bleeding, loose stools, and abdominal pain.

In 2018, the patient still had 10-15 loose stools per day with PR bleeding. Henceforth, fecal calprotectin levels were checked, which were mildly elevated, but the MRI of the small bowel and lactose tolerance test were normal. Another colonoscopy was done in August 2018, which was normal, including histology.

**Figure 1:**
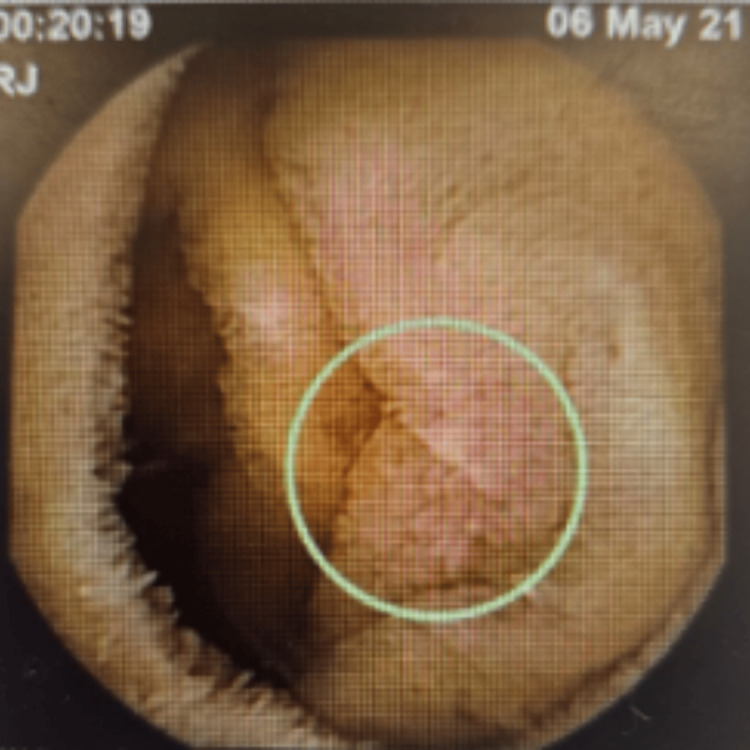
Video capsule endoscopy in 2021 showing small aphthous ulcer with erythema

**Figure 2:**
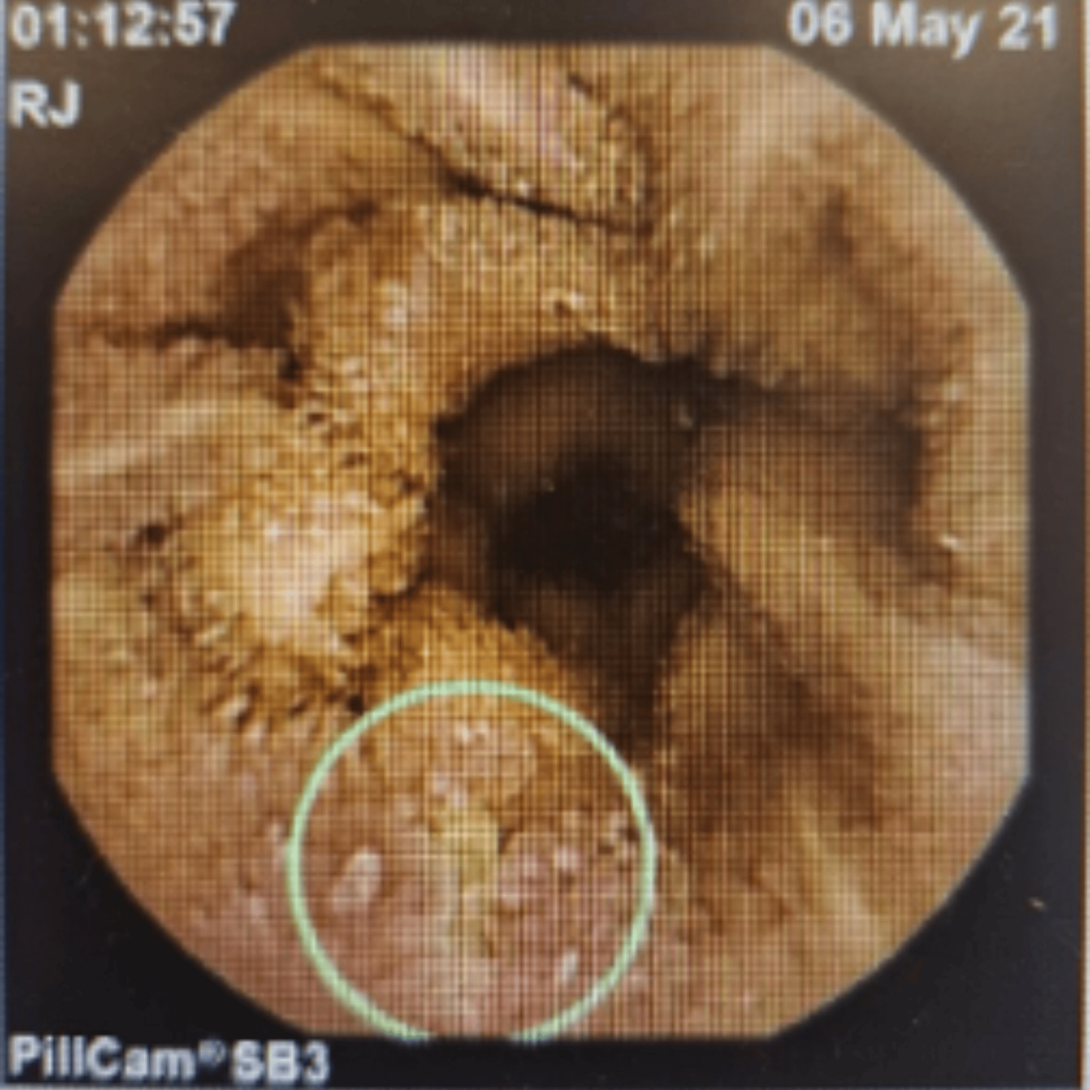
Video capsule endoscopy in 2021 showing distal small bowel aphthous ulcers

**Figure 3:**
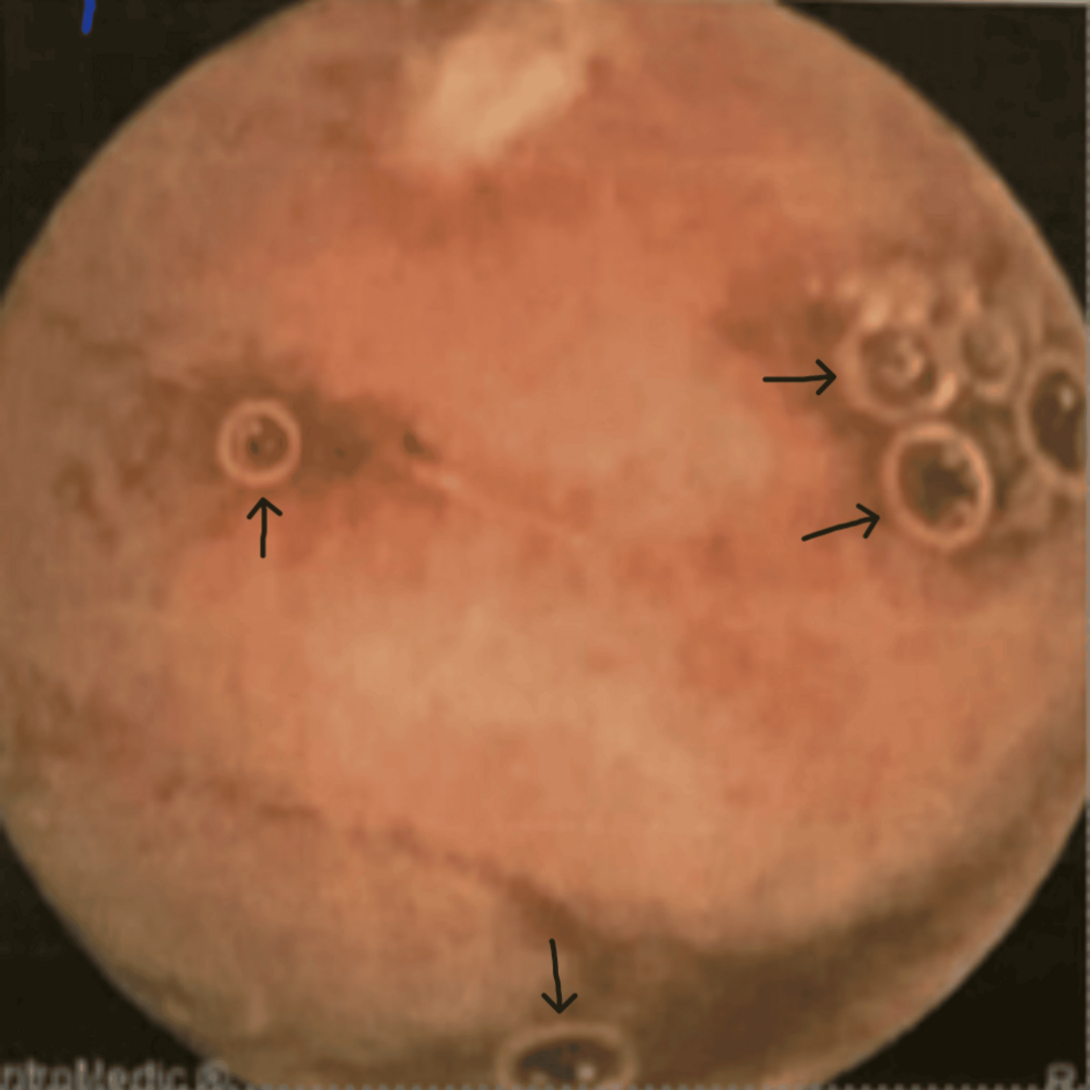
Video capsule endoscopy in 2023 showing shallow ulcers (black arrows)

In 2021, a video capsule endoscopy revealed duodenitis and multiple small and large aphthous ulcers scattered throughout the small bowel, which appeared more so in the proximal small bowel/jejunum (Figure 1), and small aphthous ulcers were also seen in the very distal ileum (Figure 2), suggesting inflammatory small bowel disease (CD). The patient was started on budesonide, followed by adalimumab in January 2022, and later switched to vedolizumab in December 2022 due to persistent symptoms of PR bleed, loose stools, and abdominal pain. Another video capsule endoscopy in 2023 showed four shallow ulcerations approximately 4 mm in size and several erosions in the jejunum with normal surrounding mucosa (Figure 3), and his treatment was changed to ustekinumab in July 2023 due to persisting symptoms as mentioned above, despite treatment with vedolizumab.

Fecal calprotectin values varied from 2022 to 2023 (Table 1).

**Table 1:** Fecal calprotectin values for the patient

Date​	Result microgram/gram​ (normal fecal calprotectin value is <50 mcg/g)
January 26, 2022	334​
July 26, 2022	1009​
October 12, 2022	1366​
October 15, 2022	>1800​
January 10, 2023	574​
August 4, 2023	132​
November 28, 2023	546​

In March 2024, the patient presented with dizziness, abdominal cramps, and bloody diarrhea (12 times a day), not responding to ustekinumab. His hemoglobin was 79 on admission, and he was treated with IV hydrocortisone and blood transfusion. A CT abdomen showed no intrabdominal pathologies. A repeat video capsule endoscopy was done in February 2024, which showed two 1 mm aphthoid erosions seen in the duodenal bulb with two superficial ulcers ranging from 3 mm to 5 mm in the duodenojejunal junction with normal surrounding mucosa.

**Figure 4:**
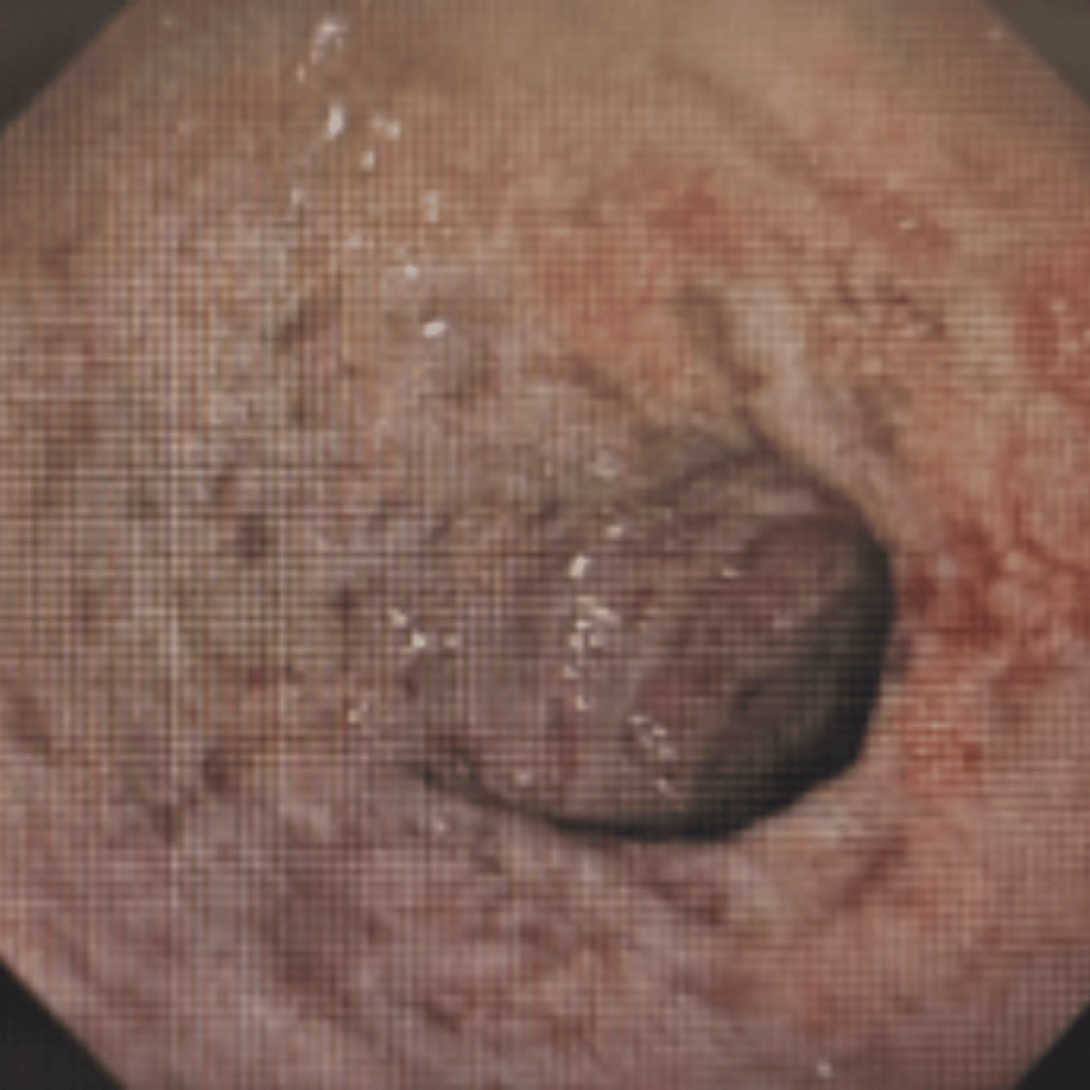
OGD in 2024 showing gastritis and erosive duodenitis OCD: esophagogastroduodenoscopy

An esophagogastroduodenoscopy (OGD) performed on March 20, 2024, revealed gastritis and erosive duodenitis, and a biopsy was taken (Figure 4).

Biopsy results

**Figure 5:**
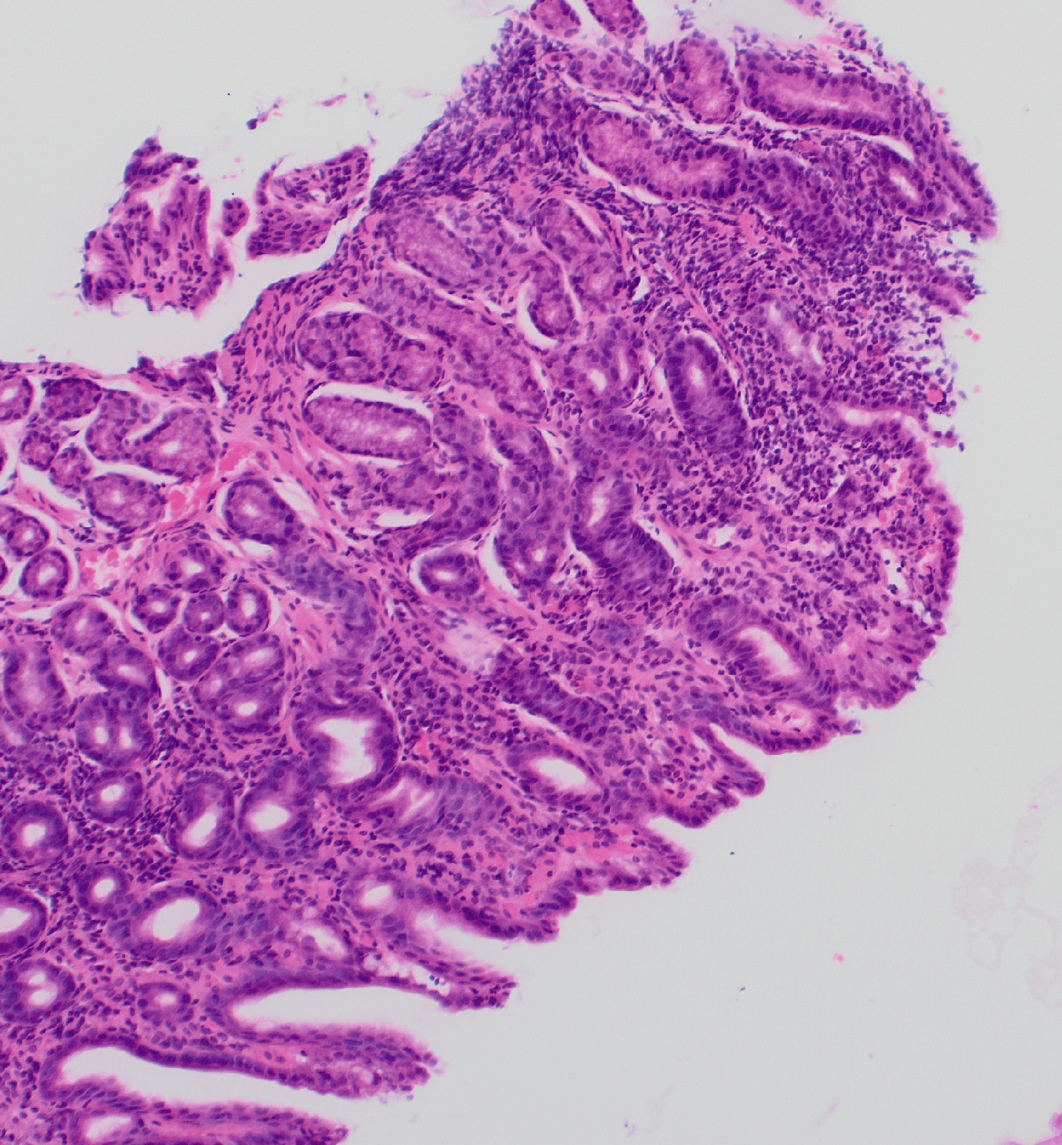
Histology picture demonstrating acute inflammation/neutrophil polymorphs in the villous and crypt epithelium

**Figure 6:**
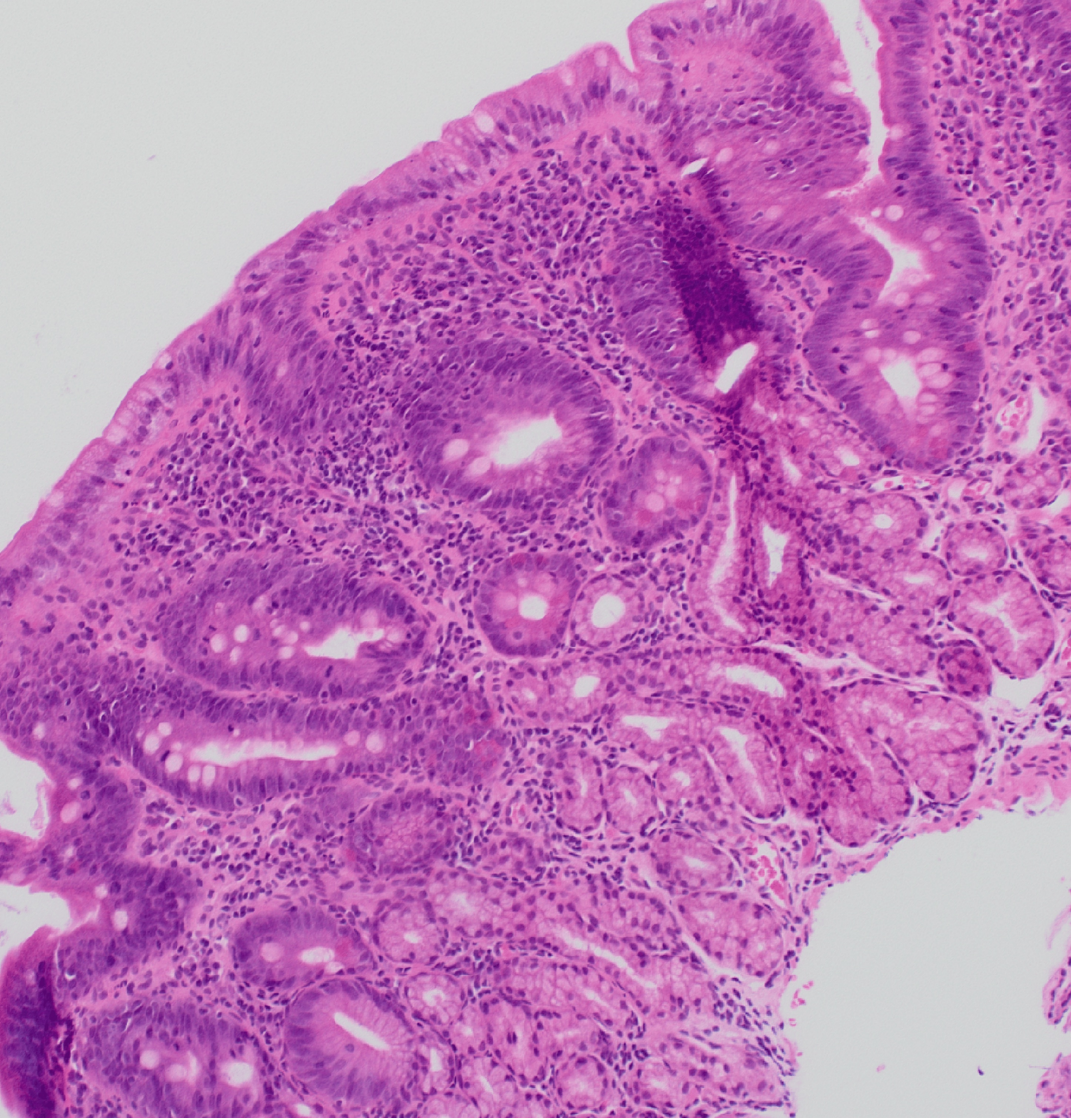
Demonstration of acute inflammation/neutrophil polymorphs in the villous and crypt epithelium

D1 biopsy showed patchy mild expansion of the villous stroma by chronic inflammatory cell infiltrate. A small number of neutrophil polymorphs are seen in the surface epithelium in one fragment, indicating active inflammation (Figures 5-6). The features are those of focal active chronic duodenitis. Active duodenitis is not specific for CD and is most commonly seen with medications such as nonsteroidal anti-inflammatory drugs (NSAIDs). It is a non-specific feature that can be seen in UGI CD. However, in this case, medications and *Helicobacter pylori* infection were ruled out, leading to a diagnosis of UGI CD. A more specific histologic finding is the presence of granulomas, but these are seen only in a small number of cases.

**Figure 7:**
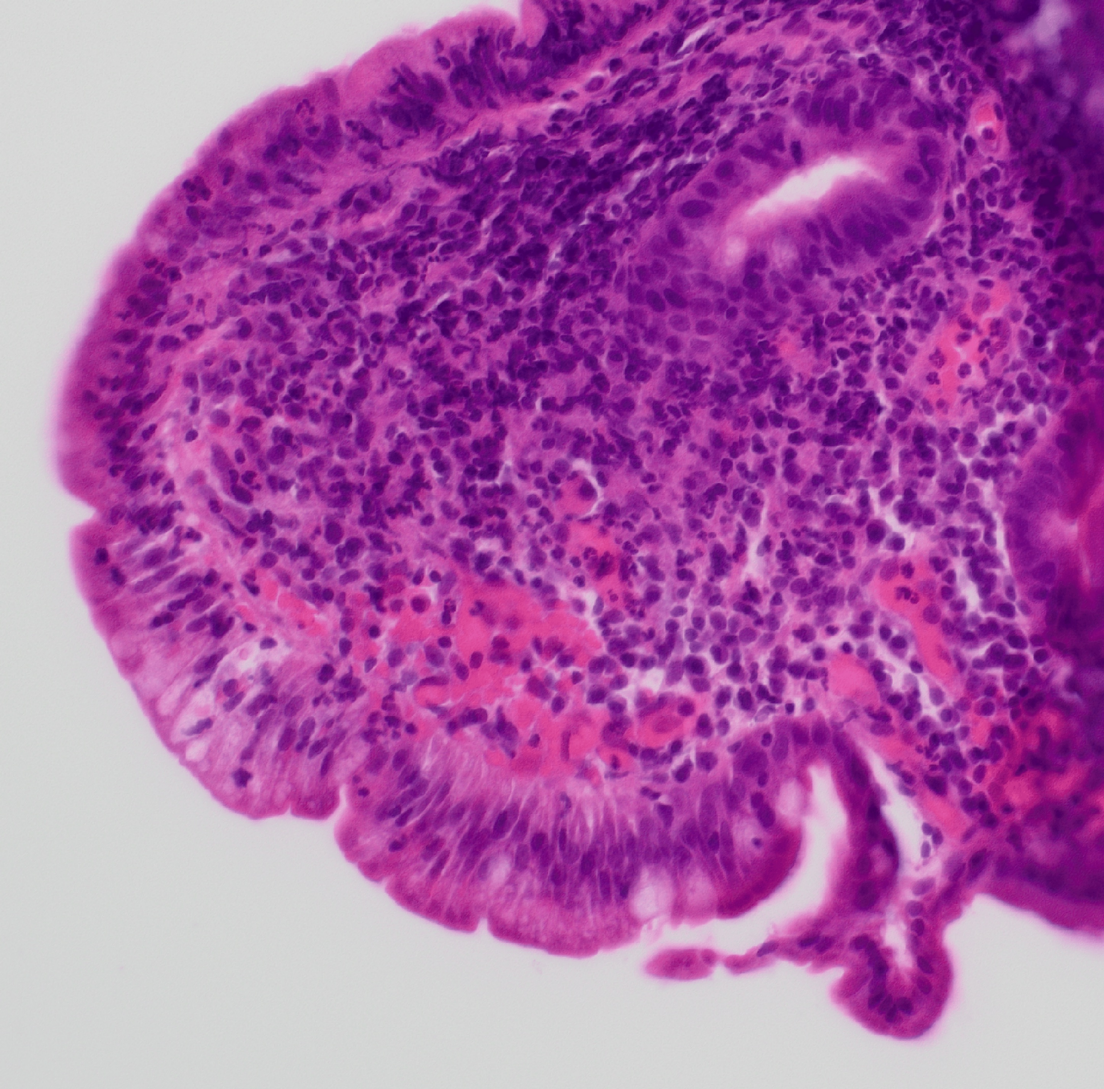
Histology picture demonstrating acute inflammation/neutrophil polymorphs in the villous and crypt epithelium

D2 biopsy showed focal mild expansion of the villous stroma by chronic inflammatory cell infiltrate; a small number of neutrophil polymorphs are seen in the lamina propria and in the surface epithelium, indicating minimal active inflammation, suggestive of focal active chronic duodenitis (Figure 7).

Gastric biopsy showed a mild to moderate chronic inflammatory cell infiltrate in the lamina propria of the antral mucosa. A small number of neutrophil polymorphs are seen on the surface and occasional crypt epithelium, indicating active inflammation. The lamina propria in the specialized gastric mucosa shows a patchy chronic inflammatory cell infiltrate. *H. pylori *was not identified in the Giemsa stain; the features are those of focal active chronic gastritis. In the absence of NSAID intake and *H. pylori* infection, this pattern of gastritis is quite characteristic of UGI tract involvement in CD in adults.

As per the histological characteristics in this literature, this would classify the above histological findings as gastro-duodenal CD.

Despite these findings, the MRI of the small bowel on April 25, 2024, showed no active CD. Symptoms persisted, leading to a change in ustekinumab frequency from eight weekly to six weekly. Another MRI of the small bowel on June 14, 2024, was also unremarkable.

The dietitian reviewed the patient in view of ongoing vomiting, and a plan for nasojejunal (NJ) insertion was suggested and was inserted on June 27, 2024.

The patient’s symptoms improved with nutritional management, and a trial of oral feeding was given. The patient tolerated oral feed, and the patient became symptomatically better; hence, he was discharged with a plan to follow up.

## Discussion

The diagnosis of CD is based on clinical symptoms, laboratory, endoscopic, histological, and radiographic findings. Adults presenting with UGI CD are considered uncommon. The prevalence in adults is thought to range between 0.3% and 5%, with an incidence of roughly 16% [4].

Clinical diagnosis of CD of the duodenum (2%-4%) and stomach (0.5%-4%) is uncommon. Gastric involvement typically develops in patients with a concurrent duodenal disease. The majority of inflammatory bowel disease (IBD) patients with UGI involvement typically either show concurrent ileocolonic disease or were previously diagnosed with lower gastrointestinal IBD [5].

Diagnosing UGI CD can be challenging due to its nonspecific symptoms and the underutilization of UGI endoscopy. CD patients with dyspepsia, abdominal pain, and vomiting benefit from a UGI endoscopy and biopsy [6].

Patients with esophageal involvement may present with symptoms such as heartburn, regurgitation, and chest pain. The endoscopic findings can be friability, erosions, aphthoid ulcers, superficial or deep ulcerations, nodularity and stiffness of the mucosa, cobblestone appearance, and inflammation. The histological findings are a chronic inflammatory infiltrate with a predominance of lymphocytes in the lamina propria and the presence of ulcers [7].

In patients with gastroduodenal involvement, endoscopic findings may include edema, enanthem, longitudinal or irregular erosions, superficial or deep ulcers (including aphthoid ulcers), a cobblestone appearance, notching of the duodenal folds, and a bamboo-joint-like appearance. The histological findings are acute and chronic inflammation, focal inflammatory changes, lymphoid aggregates, mucosal-muscular fibrosis, chronic *H. pylori *negative gastritis, focal gastritis, epithelioid granuloma, and duodenitis [7].

A prospective study on the role of upper endoscopy in the diagnostic workup of UGI involvement in CD was conducted with CD patients, who underwent clinical assessment and UGI endoscopy with biopsy samples for histological assessment and *H. pylori* infection detection. A high prevalence of upper GIT involvement was observed in CD patients, irrespective of upper symptoms. The findings highlighted the usefulness of routine UGI endoscopy and histology in the diagnostic work-up of CD patients in order to correctly classify the distribution and extent of the disease [8].

Measurement of fecal calprotectin is a useful surrogate marker of gastrointestinal inflammation. It has a high negative predictive value in ruling out IBD in undiagnosed, symptomatic patients and a high sensitivity for diagnosing the disease, making it useful as a tool for prioritizing endoscopy [9]. Fecal calprotectin is produced by neutrophils and other inflammatory cells that migrate from the gastrointestinal mucosa into the intestinal lumen during inflammation. Furthermore, fecal calprotectin has become an important tool in distinguishing between organic inflammatory diseases, such as IBD, and functional gastrointestinal disorders, including irritable bowel syndrome. At present, fecal calprotectin levels under 50 μg/g are regarded as normal. Fecal calprotectin concentrations above 200 μg/g, on the other hand, could indicate an active endoscopic state of IBD [10].

In this patient, endoscopic evaluation of the GIT was suggestive of significant UGI (gastroduodenal) CD, which was confirmed by histological evidence. This was further supported by the significant elevation of fecal calprotectin (>1800) levels.

Management of upper GIT CD

Due to the rarity of UGI CD and its frequent overlap with distal forms of the disease, there is limited data on its specific management. Consequently, treatment for UGI CD often follows the same general strategies as those used for distal disease. Corticosteroids remain the cornerstone of medical treatment for UGI CD. The Asia-Pacific consensus guidelines for CD suggest using azathioprine, 6-mercaptopurine, or methotrexate as steroid-sparing agents, though there is a lack of robust evidence supporting their effectiveness in UGI involvement. Likewise, there is insufficient data on the use of sulfasalazine and mesalazine in UGI CD. Infliximab is the most commonly utilized biologic therapy for this condition. Approximately 40-50% of patients with severe UGI CD require surgery after failing medical management. Surgical interventions like strictureplasty and bypass procedures, including gastroduodenostomy and duodenojejunostomy, are among the most commonly performed operations [11].

Several new exclusion diets modeled after exclusive enteral diet (EEN) and specific carbohydrate diet (SCD) have shown potential efficacy in smaller studies. However, there is a paucity of clear dietary recommendations for reducing clinical relapse of CD [12].

For children and adolescents with active CD, exclusive enteral nutrition (EEN) is recommended as the first-line treatment to induce remission. The CD exclusion diet (CDED) was developed to incorporate whole foods along with parenteral nutrition (PEN), while restricting or eliminating foods known to trigger inflammation, such as gluten, dairy, animal fats, processed meats, products with emulsifiers, canned foods, and packaged items. Studies have demonstrated that adherence to the CDED, with or without PEN, can help induce remission in mild-to-moderate luminal CD in both pediatric and adult populations, while also reducing inflammatory markers [13].

## Conclusions

This case highlights the complexity of diagnosing and managing UGI CD. Despite multiple endoscopic and imaging evaluations, the patient’s diagnosis remained challenging. UGI involvement in CD requires a high index of suspicion and thorough endoscopic and histological evaluations to guide appropriate treatment and management. This case also highlights the importance of pathology in UGI CD, even when endoscopy is normal, if there is clinical suspicion. Fecal calprotectin is an effective biomarker for the severity of CD and an indication of ongoing inflammation even in the presence of unremarkable imaging. Additionally, this case also highlights the effective role that nutrition management plays in CD.
